# Seasonal variability of the microzooplankton biomass and community composition on the Northeast US Shelf

**DOI:** 10.1093/plankt/fbag018

**Published:** 2026-03-28

**Authors:** Frankie Lopez, Pierre Marrec, Susanne Menden-Deuer

**Affiliations:** Graduate School of Oceanography, University of Rhode Island, Narragansett, RI, 02882, USA; Graduate School of Oceanography, University of Rhode Island, Narragansett, RI, 02882, USA; Graduate School of Oceanography, University of Rhode Island, Narragansett, RI, 02882, USA

**Keywords:** microzooplankton, continental shelf, microzooplankton grazing, planktonic food web, long term ecological research

## Abstract

Grazing on phytoplankton by microzooplankton is a critical process in making energy available to higher trophic levels, shaping marine food webs, and biogeochemical cycling. Despite its key role, the drivers of grazing remain poorly constrained due to the concurrent influences of biotic and abiotic factors. To elucidate the impact of microzooplankton biomass and community composition on grazing rates, we measured ciliate and dinoflagellate biomass in boreal summer and winter from 2018-2023, at three stations spanning the Northeast US Shelf from coastal Rhode Island, USA to the shelf break. This record of microzooplankton biomass encompasses the seasonal and spatial variability from shore to shelf. Dinoflagellate biomass showed a clear seasonal shift from larger (>20 μm) cells in winter to smaller ones in summer, while ciliate biomass lacked seasonal trends. Anomalies in community composition coincided with disruptions in the typical seasonal phytoplankton size structure, suggesting that microzooplankton size structure was linked to the size of available prey. In winter, microzooplankton biomass was positively correlated with microzooplankton grazing, indicating that the mechanism moderating the planktonic food web structure changes seasonally. These results inform our understanding of planktonic food webs by identifying the linkage between grazing and seasonal changes in phytoplankton community structure that impact energy transfer to higher trophic levels.

## INTRODUCTION

Microzooplankton are the primary consumers of phytoplankton across the world’s surface ocean (Calbet and Landry, 2004). Microzooplankton biomass is mostly composed of heterotrophic protists, such as ciliates and dinoflagellates, less than 200 μm in size. Most microzooplankton consume phytoplankton via phagotrophy in a process known as grazing (Kirchman, 2018), and many are known to be mixotrophic (Stoecker *et al.,* 2017). Microzooplankton are critical components of planktonic food webs and biogeochemical cycles, grazing up to 67% of total primary production in the surface ocean (Calbet and Landry, 2004; Schmoker *et al.,* 2013), serving as vectors of energy, nutrients, and organic matter to higher trophic levels, while also playing a role in the remineralization of organic matter and nutrients (Landry *et al.,* 2024). While grazing is highly influential on marine food webs and biogeochemical cycles, limited empirical data on microzooplankton biomass and grazing rates limits our understanding of their role in these processes (Schmoker *et al.,* 2013). Grazing remains the highest contributor to uncertainty in estimates of carbon sequestration and trophic transfer in global biogeochemical models (Rohr *et al.,* 2023) and a mechanistic understanding of the drivers of grazing will allow for more robust predictions of these critical parameters.

The Northeast US continental shelf (NES) is a temperate coastal ecosystem off the east coast of the United States, known for its high ecological and economic productivity (Hoagland *et al.,* 2005; Fontaine *et al.,* 2025). This dynamic ecosystem is under the influence of variable hydrological conditions due to the warm, salty Gulf Stream flowing north offshore and the Labrador Current carrying cold and fresher water south along the coast (Csanady and Hamilton, 1988; Chapman and Beardsley, 1989). The interaction of these two water masses leads to a shelf-break front on the edge of the continental shelf. During winter, strong mixing and terrestrial input of nutrients support large, bloom-forming phytoplankton that grow slowly (Yoder *et al.,* 2002). Summer is characterized by depleted nutrient stocks and strong stratification of the surface ocean which favors the fast growth of small phytoplankton (Fowler *et al.,* 2020). These seasonal changes in the phytoplankton community structure have cascading effects on the function of the entire marine food web. The strong environmental gradients on the shelf, both seasonally and regionally, make it an ideal study site to investigate how the planktonic food web is influenced by the environment.

Surprisingly, while much of the planktonic food web is strongly seasonal on the NES, grazing rates of microzooplankton do not reflect these patterns and rates appear to vary little over time and space (Marrec *et al.,* 2021). With comparable microzooplankton grazing rates across seasons, slow phytoplankton growth rates in winter lead to a larger fraction of primary production being consumed by microzooplankton grazing than in summer when higher phytoplankton growth rates are reported (Marrec *et al.,* 2021). To our knowledge, there are no records of the abundance and biomass of microzooplankton for the NES. These measurements are time-consuming and require substantive expertise, so microzooplankton biomass estimates are often reported from single cruises, with limited temporal and spatial coverage. Even in the case that microzooplankton biomass and grazing are measured concurrently, it is still challenging to determine the drivers of grazing intensity (Lawrence and Menden-Deuer, 2012). Here we report estimates of microzooplankton biomass for two key groups of microzooplankton on the NES, dinoflagellates and ciliates, in winter and summer from 2018-2023 along with a comprehensive set of environmental, chemical, and biological conditions. These estimates are paired with grazing rates measured by the dilution method and span a shore to shelf transect of over 100 nautical miles, allowing us to assess how the observed grazing rates are related to the composition, size-structure, and total biomass of the predator community under a broad range of environmental conditions.

## METHODS

### Study site and sample acquisition

This research was done in the context of the Northeast US Shelf Long Term Ecological Research program (NES-LTER). Microzooplankton biomass samples were collected on twelve NES-LTER seasonal transect cruises, eleven on board the *R/V Endeavor*, and one on board the *R/V Atlantis* in each boreal winter (January or February) and summer (July or August) from 2018-2023. The set of samples analyzed here were collected at three locations along the NES-LTER transect, one on the inner shelf (<50 m depth), one on the mid-shelf (50-100 m depth), and one on the outer shelf (>100 m depth) at stations on the NES-LTER transect. The division of the shelf into bathymetry-based regions follows the approach of previous studies that have shown that this partitioning effectively delineates areas with distinct physical and biogeochemical characteristics (Castillo Cieza *et al.,* 2024; Fontaine *et al.,* 2025). In keeping with the station designations of the NES-LTER, these stations are L1, L4, and L11. Station locations can be found in Marrec *et al.* (2021). All samples reported in this study were collected from surface waters.

Water was collected from Niskin bottles mounted on a CTD-rosette system (Seabird SBE32 Carousel Water Sampler). A silicone tube fitted with 200 μm mesh was used to remove mesozooplankton and gently transfer water from the Niskin into a 10 L carboy which was also used to prepare microzooplankton grazing experiments previously reported by Menden-Deuer and Marrec (2023) and outlined below. Microzooplankton biomass samples were collected from these carboys after thorough and gentle mixing. Approximately 300 mL of seawater were gently transferred into an amber glass jar. Samples were fixed using 2% Lugol’s acid (Menden-Deuer *et al.,* 2001) and stored in the dark until analysis in the lab. While preservation in Lugol’s can introduce some species-specific changes in cell size, it has been shown that it does not introduce directional bias when assessing mixed communities (Menden-Deuer *et al.,* 2001).

### Environmental variables

At each station, temperature and salinity profiles were measured throughout the water column using a SBE911 CTD (Seabird Electronics). Additional sensors were used to measure chlorophyll-a (Chl-a) fluorescence and photosynthetically active radiation (PAR, Biospherical Instruments® QSP2000). Dissolved inorganic nutrients (nitrate+nitrite, ammonium, phosphate, and silicate) were sampled from Niskin bottles on the CTD-rosette and measured by the WHOI Nutrient Analytical Facility using a SEAL Analytical AA3 HR. More details about methods for measurement of dissolved nutrient concentrations can be found in Sosik et al. (2025). Estimates of surface PAR (mol photons m^−2^ d^−1^) were retrieved from 8-d satellite MODIS Aqua PAR L3 products (https://oceancolor.gsfc.nasa.gov/). Light attenuation coefficients (K_d_) were calculated from CTD profiles of PAR during daytime casts and were estimated from linear correlations between K_d_ and mean beam attenuation between 0 m and 10 m depth for nighttime casts (Marrec *et al.,* 2021). Mixed layer depth (MLD) was determined as the shallowest depth corresponding to a temperature difference with the surface water greater than 0.5°C (Monterey & Levitus 1997). The mean light intensity in the mixed layer (I_m_) was calculated using surface PAR, MLD, and K_d_ (Domingues and Barbosa, 2023).

### Chlorophyll-a concentration

Total and size-fractionated Chl-a concentrations were measured as a proxy for phytoplankton biomass. In situ Chl-a concentration was measured from the 10 L carboys for both dilution levels described below at the time of sampling. Triplicate 152 mL samples were filtered using GF/F ($>$0.7 μm) and polycarbonate membrane filters ($>$10 μm). Filters were extracted in 95% ethanol in the dark at room temperature for 12 hours before fluorescence was measured on a calibrated Turner 10AU fluorometer using the acidification method (Wasmund *et al.,* 2006). The difference in Chl-a concentration between the $>$10 μm and GF/F filters was used to calculate the Chl-a concentration of the $<$10 μm size fraction.

### Phytoplankton growth and microzooplankton grazing rates

Here we use previously published microzooplankton grazing rates to assess their relationship to microzooplankton biomass and community composition (Menden-Deuer and Marrec, 2023; Marrec and Menden-Deuer, 2024), and methods used are detailed there. In brief, phytoplankton growth rates and rates of mortality due to microzooplankton grazing were measured using the 2-point modification of the dilution experiment (Landry *et al.,* 2008; Chen, 2015; Morison and Menden-Deuer, 2017). At each station, whole seawater from the surface (WSW) was sampled from the Niskin, screened with 200 μm mesh, and gently transferred into a 10 L carboy using a silicone tube. Diluent was prepared by gravity filtration directly from the Niskin using a 0.2 μm membrane filter capsule (PALL) and combined with WSW in a 10 L carboy to obtain a 20% dilution level. Diluent was prepared from the same depth as the WSW. Each dilution experiment consisted of 6 × 1.2 L polycarbonate bottles, duplicates of both the WSW and 20% diluted treatments with added nutrients and duplicates of WSW with no added nutrients, to test for nutrient limitation. Starting in summer 2022, the addition of triplicate nutrient-amended bottles yielded 8 bottles total. Bottles were incubated for 24 h in deck-board incubators with flow-through seawater to maintain ambient temperature and placed in mesh bags to replicate the light level in the surface mixed layer. Chl-a concentration was measured at the end of the incubation in triplicate 152 mL subsamples from each incubation bottle using GF/F filters according to the methods outlined above. In the case that the growth rates in diluted and non-diluted treatments were not significantly different from each other, the grazing rate is considered to be zero. In the case that growth is higher in the non-diluted treatment than the diluted treatment, grazing is considered to be “undetermined”. Seven out of the 36 dilution experiments coinciding with the measurements of microzooplankton biomass presented here resulted in undetermined grazing rates, yielding 29 experiments with measurable grazing rates.

### Microscopy and image analysis

In the lab, between 10 and 50 mL of each Lugol’s fixed sample were settled into a 1 mL sedimentation slide with an Utermöhl chamber (HYDRO-BIOS, Utermöhl, 1958) and analyzed using a Nikon E800 inverted microscope with a magnification of 4x, 10x, 20x, or 40x. Images were taken using a mounted Stingray (model F-146) or OMAX (model A35180U3) camera. Both cameras were calibrated with a micro ruler at each magnification level to convert pixels to micrometers.

A semi-automated image analysis process was used to convert raw microscope images to estimates of carbon biomass. The contrast of the images was adjusted to distinguish regions of interest (ROI, e.g. particles), and the ROIs were automatically cropped into a single image file. All cropped pictures were then checked manually to determine which ROIs represented microzooplankton. The *regionprops* MATLAB function was used to automatically measure the major and minor axes lengths of each cell. The initial measurements for ciliate major axes are overestimates, since the automated measurements include the cilia which do not contribute substantially to carbon biomass. Therefore, a subset of 215 ciliates were manually measured in ImageJ excluding the cilia. The regression between manually and automatically measured lengths revealed that cilia contributed on average 12% to the overall length of ciliates, which would lead to an overestimation using the automated measurements. This relationship was used to determine a correction factor that was applied to all ciliates in the dataset, and therefore this overestimation is corrected for in the data reported here.

Biovolume was calculated from size measurements of 2D images, assuming all cells are prolate spheroids (Hillebrand *et al.,* 1999). Estimates of biomass calculated from 2D measurements of cell images do not differ significantly from 3D estimates of volume (McNair *et al.,* 2021). Carbon biomass was calculated from biovolume using established carbon to volume relationships from Menden-Deuer and Lessard (2000). Here we report biomass estimates of dinoflagellates and ciliates. The use of an inverted microscope to image Lugol’s preserved samples precludes the differentiation of dinoflagellates containing plastids or chlorophyll, and therefore, some autotrophic dinoflagellates were included in these estimates. However, the trophic status of many dinoflagellates previously believed to be strictly autotrophic has been revised in recent years as it becomes more apparent that mixotrophy is prevalent amongst marine dinoflagellates (Jeong *et al.,* 2010; Flynn *et al.,* 2013; Jones *et al.,* 2025). Many plastid- and chlorophyll-containing dinoflagellates are still capable of heterotrophy and should therefore be considered as potential grazers (Stoecker *et al.,* 2017). Cell size for large dinoflagellates of the genus *Tripos* were measured manually and biomass was calculated based on morphotype (Napiórkowska-Krzebietke and Kobos, 2016). The presence of *Tripos* was sporadic and highly influential on biomass, so they are excluded from estimates of overall dinoflagellate biomass reported here and instead are presented separately (Table S1). Dinoflagellates and ciliates were manually identified. Values reported here are for two size categories, a major axis length of greater and less than 20 μm, respectively. Total number of cells imaged per sample depended on cell abundance and ranged from 41 to 456, with less than 80 images in 5 out of 36 samples. The number of dinoflagellate cells imaged per sample ranged from 25 to 423, and ciliate cells imaged per sample ranged from 2 to 79. The smallest cells identified for both dinoflagellates and ciliates were approximately 4-5 μm in length, setting the lower limit for the <20 μm size class. Due to their small size, heterotrophic nanoflagellates (HNF) are excluded from estimates of microzooplankton biomass reported here. However, we expect their contribution to the total heterotrophic protist biomass to be small, even if they are present in high abundances, due to their small cell size (Levine *et al.,* 2025).

### Statistical analysis

All statistical analyses were performed in MATLAB using the Fathom toolbox (Jones, 2008). A significance level of *p*<0.05 was used for all statistical tests. Relative biomass in all instances was calculated as the proportion of the biomass of one of the four groups of microzooplankton (ciliates and dinoflagellates in two size classes each) to the sum of all four groups. Nutrient concentrations were considered zero for all statistical analyses, including reported means, when they were below the detection limit. Analyses of significant differences of the overall community composition between season and region were performed iteratively 999 times via ANOSIM (f_anosim) on a Bray-Curtis (f_dis) dissimilarity matrix of the absolute biomass of each of the four microzooplankton groups. Analysis of significant seasonal and regional differences of individual microzooplankton groups and the total microzooplankton biomass were performed using a two-way ANOVA with a type III sum of squares and with outlier events excluded.

The redundancy analysis (RDA) was performed using a subset of the environmental variables (nitrate+nitrite, MLD, and salinity), all normalized by z-score, and the relative biomass of the four microzooplankton groups (f_rda). Co-correlation was assessed using Pearson’s correlation coefficient (r) and *p*-value, with correlations being considered significant when r>0.70 and *p*<0.05. Co-correlations above these thresholds were used to eliminate factors used in the RDA to only include independent environmental variables. The RDA was performed iteratively 999 times. We explored several different environmental parameters to identify which metrics were both meaningful in explaining the conditions relevant to the microzooplankton community, while also allowing for statistically robust analysis by minimizing co-correlation between independent variables. Several of the environmental variables were significantly correlated with one another (Figure S1). Temperature was significantly correlated with I_m_, PAR, Chl-a, and the percentage of Chl-a smaller than 10 μm. Of these variables, percentage of Chl-a smaller than 10 μm had the highest cumulative correlation to the four microzooplankton groups and therefore was included in the redundancy analysis while its co-correlates were excluded. We included one variable to represent nutrients (nitrate+nitrite) and one to represent light availability (MLD), two essential resources for phytoplankton growth. These two variables were not co-correlated with any other environmental variables, and they had the strongest correlation with microzooplankton biomass compared to the other nutrients (phosphate, silicate, ammonium) and light variables (PAR, I_m_, Kd). Salinity was not co-correlated with any other environmental parameter and was therefore included in the RDA. Salinity can be used as an indicator of spatial variability along the shelf, as it shows a similar coast-to-offshore gradient in both winter and summer.

To detect outliers in microzooplankton community composition, we calculated the Euclidian distance of each data point from the centroid of both seasons in RDA-space. Points were considered outliers if they were more than 50% closer in Euclidian distance to the centroid of the opposite season, or if they were more than 2.5 standard deviations from the centroid of their own season. The three outlier observations were from winter of 2020 on the outer-shelf, summer of 2018 on the outer-shelf, and summer of 2019 on the mid-shelf. These outliers were not included in any reported mean values. However, as these outliers represent notable events, they are discussed separately.

## RESULTS

### Environmental conditions

Environmental conditions on the shelf varied by region and season (Table S2). Temperature increased from the coast to offshore in both winter and summer, averaging 4-12 and 21-25 °C from the inner- to the outer-shelf, respectively. Salinity was similar across seasons and was on average 32-34 from the inner- to outer-shelf. Chl-a concentration decreased from on average 3.61 μg L^−1^ on the inner-shelf to 1.87 μg L^−1^ on the outer-shelf in winter, and 0.92 μg L^−1^ on the inner-shelf to 0.11 μg L^−1^ on the outer-shelf in summer. The proportion of Chl-a concentration made up of small phytoplankton (< 10 μm) was much higher in summer, on average 82%, than in winter (30%) when the phytoplankton community was composed of mostly larger cells.

In winter, nitrate+nitrite concentration increased from the inner-shelf (0.40 ± 0.88 μmol L^−1^) to the outer-shelf (4.14 ± 1.72 μmol L^−1^, Table S2). Across the shelf, nitrate+nitrite concentration was below the detection limit in summer, except once at station L11 during summer 2021 (0.29 μmol L^−1^). Phosphate concentration was relatively consistent across the shelf in winter (0.34 ± 0.13 μmol L^−1^). In summer, phosphate concentration decreased from the inner-shelf (0.16 ± 0.07 μmol L^−1^) to the outer shelf where it was below the detection limit except for in 2018 and 2020, where the concentrations were 0.09 and 0.02 μmol L^−1^ respectively. In winter, silicate concentration increased from the inner-shelf (0.57 ± 0.78 μmol L^−1^) to the outer-shelf (2.16 ± 0.75 μmol L^−1^). In summer the gradient was reversed, and silicate decreased from the inner-shelf (1.31 ± 1.20 μmol L^−1^) to the outer-shelf (0.48 ± 0.22 μmol L^−1^).

Surface PAR was relatively consistent across the shelf during both seasons and was around 3 times higher in summer than in winter (Table S2). Mixed layer depth increased from the coast to offshore in both summer and winter, and was 3, 6, and 8 times deeper in winter than summer for the inner-, mid-, and outer-shelves, respectively. Meanwhile K_d_ decreased from the coast to offshore. Reflecting seasonally higher light availability, K_d_ was higher in winter than summer for all regions. Due to the shallower mixed layer, lower light attenuation, and higher incident light, the mean light intensity (I_m_) was one order of magnitude higher in summer than in winter. I_m_ increased from the coast to offshore during summer and was similar across the shelf in winter.

### Seasonal and regional patterns of microzooplankton community structure

**Fig. 1:**
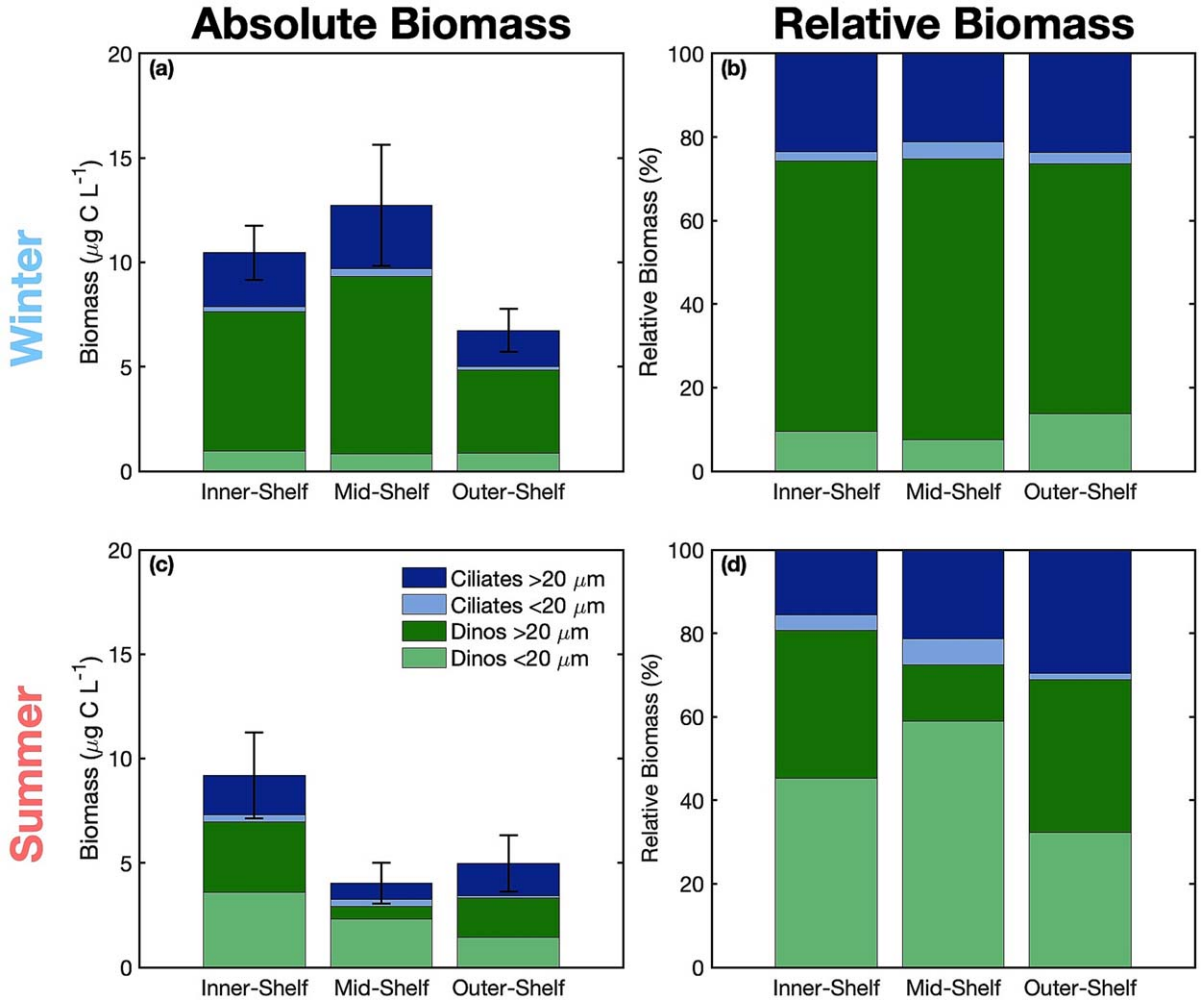
Mean absolute microzooplankton biomass (μg C L^−1^) by region for winter (a) and summer (c). Colors of stacked bars represent dinoflagellates (green) and ciliates (blue), and the two size classes, <20 μm (light) and >20 μm (dark). Error bars represent one standard error of the mean total biomass of all four microzooplankton groups. Mean relative microzooplankton biomass expressed as a proportion of each group to the total of all four groups by region for winter (b) and summer (d).

Total microzooplankton biomass was on average 65% higher in winter than in summer (*p*<0.05) (Fig. 1a and 1c). In winter, total microzooplankton biomass was highest on the mid-shelf (13 ± 7 μg C L^−1^), and lowest on the outer-shelf (7 ± 3 μg C L^−1^). In summer, highest biomass was observed on the inner-shelf (9 ± 5 μg C L^−1^), and lowest on the mid-shelf (4 ± 2 μg C L^−1^). The strongest seasonal difference was observed on the mid-shelf, with an average total biomass 3-fold higher in winter than summer. The smallest seasonal difference was observed on the outer-shelf, which on average had just 30% higher total biomass in winter than in summer.

Dinoflagellates contributed most (> 50%) of the biomass in all seasons and regions (Fig. 1b and 1d) except once (winter 2020 on the outer-shelf). On average, dinoflagellates contribute 74% of total biomass. Biomass of dinoflagellates > 20 μm in size, referred to hereafter as “large dinoflagellates,” was significantly higher in winter than summer (*p*<0.001) and was on average highest on the mid-shelf during winter (8 ± 5 μg C L^−1^), which corresponds to both the highest dinoflagellate biomass as well as the highest total biomass. The lowest large dinoflagellate biomass on average occurred on the mid-shelf during summer (1 ± 1 μg C L^−1^), which corresponds to the lowest dinoflagellate biomass as well as the lowest total biomass observed overall. The only group with significantly higher biomass in summer than winter was dinoflagellates < 20 μm in size, referred to hereafter as “small dinoflagellates” (*p*<0.001).

Ciliate biomass had no clear seasonal patterns, and ciliates consistently contributed less biomass than dinoflagellates. Biomass of small (< 20 μm) ciliates was very low (≤1 μg C L^−1^) across seasons and regions. Large (> 20 μm) ciliates contributed a relatively consistent biomass across seasons and regions, averaging 2 ± 2 μg C L^−1^ and 22% of total biomass. Relative contribution of ciliates to the total biomass was approximately 26% across shelf regions in winter and increased from coastal (19%) to offshore in summer (31%). There were no significant differences in small, large, or total ciliate biomass between seasons or regions (*p*>0.05).

The overall community composition for microzooplankton varied seasonally, with large dinoflagellates dominating in winter and small dinoflagellates contributing a greater fraction in summer. This difference was significant when considering all regions together (*p*<0.001). However, this significant seasonal difference was not observed on the outer-shelf (*p*>0.05) when each region was considered individually, suggesting that the seasonal variation was driven by changes on the inner and mid-shelf. There was no significant difference in community composition amongst the inner, mid, and outer-shelf in summer, winter, or when considering both seasons together (*p*>0.05).

### Environmental drivers

**Fig. 2:**
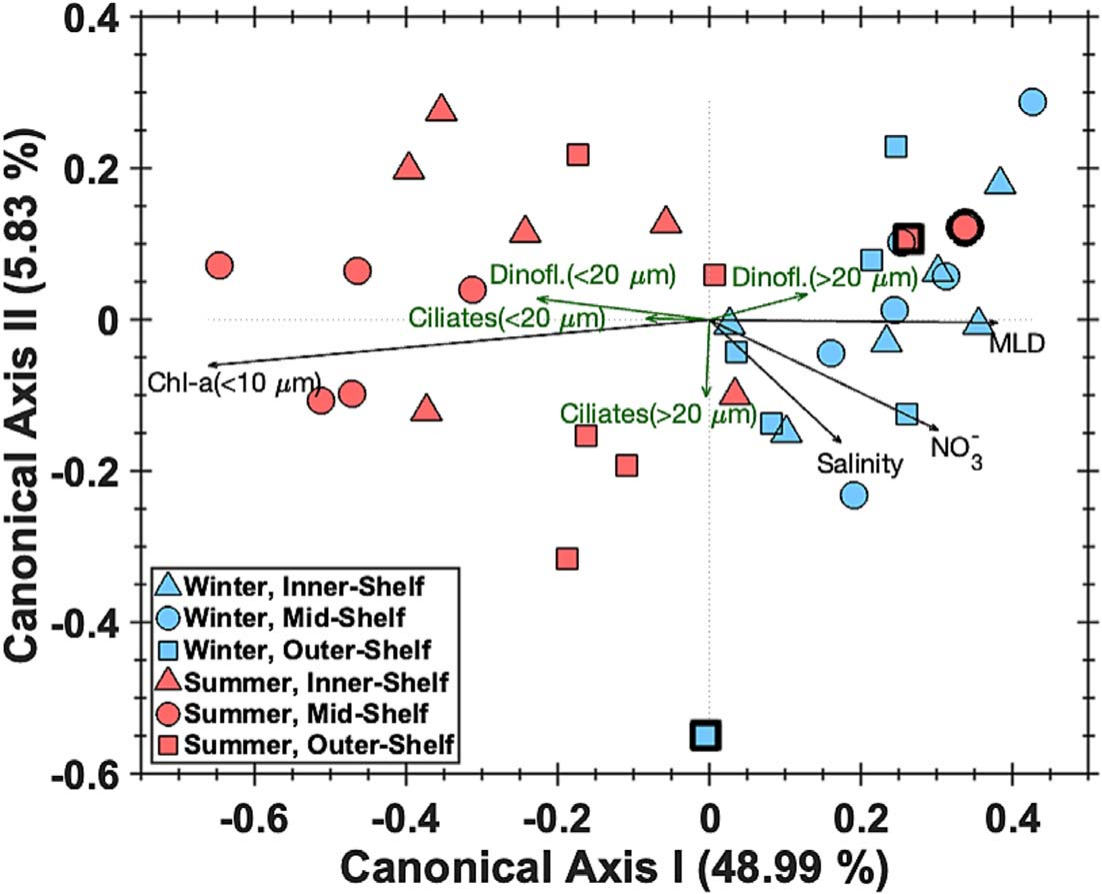
Redundancy analysis (RDA) with the relative biomass of the four microzooplankton groups as the response variables and environmental parameters (salinity, mixed layer depth (MLD), nitrate+nitrite concentration, and proportion of Chl-a contributed by phytoplankton smaller than 10 μm) as the explanatory variables. Explanatory variables are normalized by z-score before analysis. Data points are colored by season with red representing summer and blue representing winter. Markers indicate regions with triangles for the inner-shelf, circles for the mid-shelf, and squares for the outer-shelf. Enhanced outlines indicate outlier events, which are summer of 2019 on the mid-shelf, summer of 2018 on the outer-shelf, and winter of 2020 on the outer-shelf.

A redundancy analysis (RDA) was performed to determine whether variability in the environmental conditions explains variability in the microzooplankton community composition. The explanatory variables included in the RDA (fraction of small Chl-a, salinity, nitrate+nitrite, and MLD) constrain a total of 57% of the total variability observed in the microzooplankton community composition (Fig. 2). The primary axis corresponds most strongly to the fraction of Chl-a that is made up by small (<10 μm) phytoplankton, and the MLD. Salinity and nitrate+nitrite contribute to both axes 1 and 2.

### Outlier events

**Fig. 3:**
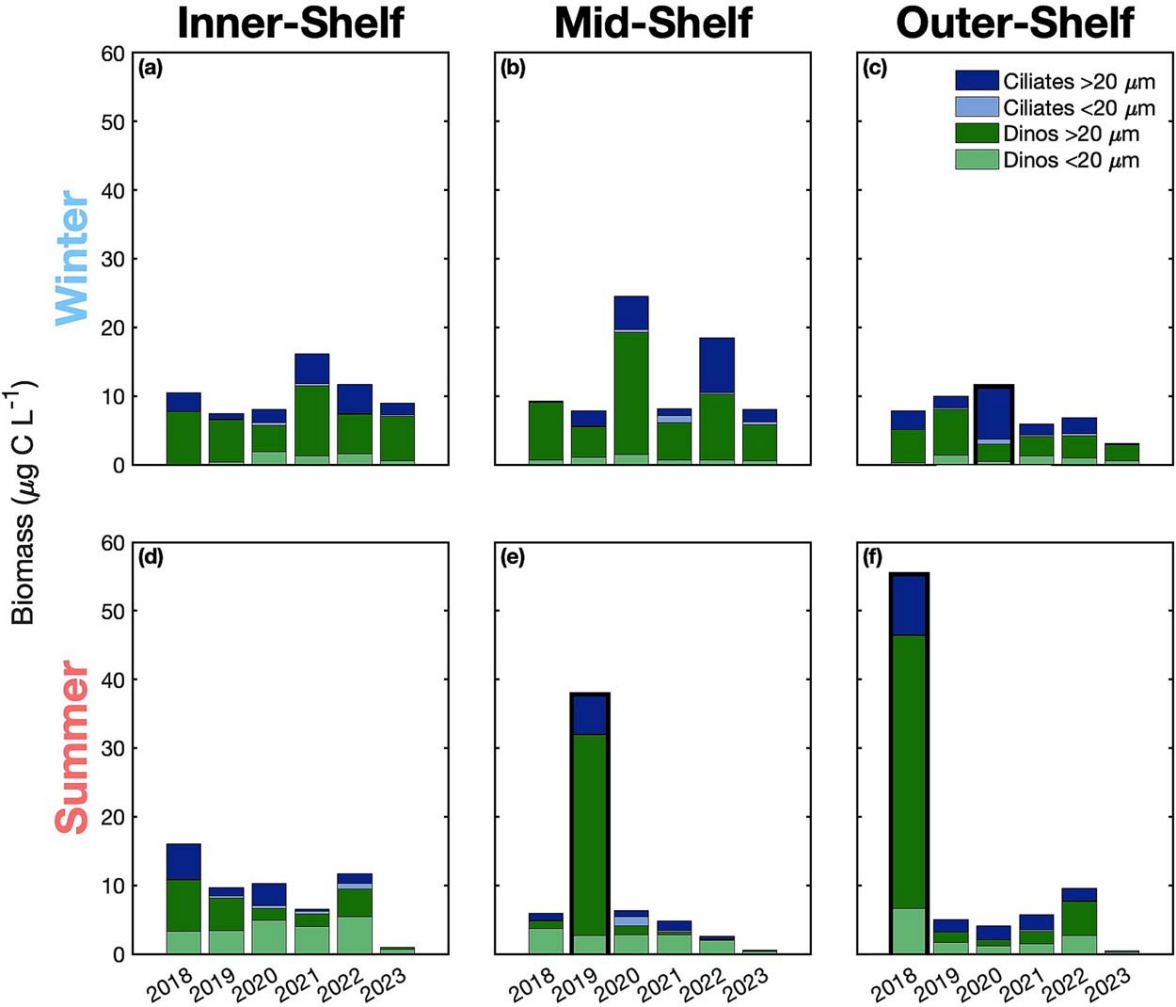
Microzooplankton biomass (μg C L^−1^) by season and region from 2018-2023. Colors of stacked bars represent the two microzooplankton groups, dinoflagellates (green) and ciliates (blue), and the two size classes, <20 μm (light) and >20 μm (dark). Bolded outlines indicate outlier events.

The redundancy analysis revealed three significant outlier events (Fig. 2). Two of these outlier events were marked by high biomass: summer of 2018 on the outer-shelf (Fig. 3f) and summer of 2019 on the mid-shelf (Fig. 3e), with total biomass 11- and 9-fold higher than the average of their respective season and region. The third outlier event, winter of 2020 on the outer-shelf had the highest proportion of ciliates compared to dinoflagellates, with ciliates contributing 73% of the total biomass, compared to the average of 25 $\pm$ 12% for all other samples (Fig. 3c).

Summer of 2018 on the outer-shelf had typical temperature, salinity, nutrient concentrations, and light conditions, with Chl-a 2-fold higher than average, and the fraction of small Chl-a 30% lower than average (Table S3). In summer of 2019 on the mid-shelf, temperature and salinity remained within typical seasonal ranges, but surface Chl-a concentrations reached 3.07 μg L^−1^, eight times the seasonal average. Winter of 2020 on the outer-shelf had typical environmental conditions, but Chl-a concentration was 43% lower than average with 73% contribution from small cells. This dominance of small cells was accompanied by a mixed layer depth 8-fold shallower than average.

### Community composition and grazing rates

**Fig. 4:**
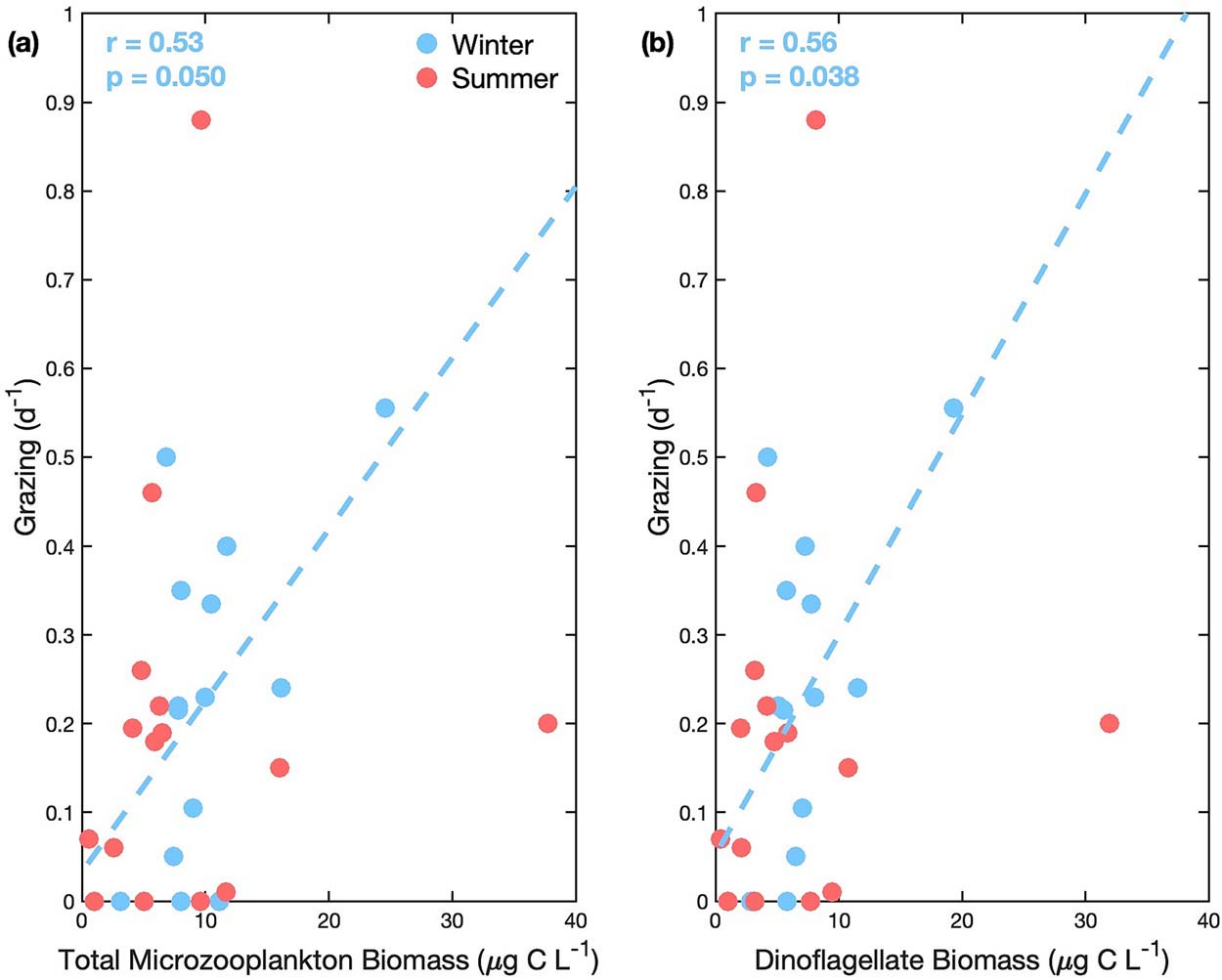
Scatterplot of the relationship of the grazing rate with (a) total microzooplankton and (b) dinoflagellate biomass (μg C L^−1^). Data points are colored by season for winter (blue) and summer (red). Regression line and statistics are shown only for winter (not significant in summer or with both seasons combined).

In winter, grazing rates had a significant positive correlation with total microzooplankton biomass (r = 0.53, *p*<0.05; Fig. 4). Grazing was also significantly correlated with the biomass of small dinoflagellates (r=0.56, *p*<0.05) and the total dinoflagellate biomass (r=0.56, *p*<0.05) in winter (Figure S2b). Grazing was not correlated to total biomass nor the biomass of any microzooplankton group in summer, or when winter and summer were combined (Figure S2a, S2c).

## DISCUSSION

The importance of microzooplankton as grazers of phytoplankton is well recognized, but the understanding of what drives the occurrence and magnitude of grazing is still limited (Steinberg and Landry, 2017). Concurrent measurements of microzooplankton biomass and grazing require expertise and are time consuming, and therefore not often measured repeatedly across time and space. This makes our dataset a valuable resource specifically for understanding food web dynamics in the NES, but also for generating testable hypotheses to unravel the complex and often puzzling nature of planktonic food webs more broadly. This study reports measurements of microzooplankton carbon biomass across a broad range of environmental conditions. We identified that dinoflagellate biomass was generally greater than ciliate biomass, and a seasonal shift from large to small dinoflagellates. Departures from the baseline patterns of microzooplankton biomass and community composition coincided with departures from the established patterns for phytoplankton, such as bloom conditions and size structure reversals (Marrec *et al.,* 2021; Marrec and Menden-Deuer, 2024). We demonstrate that examining the biomass and structure of the microzooplankton community reveals its relationship with phytoplankton size structure and conditionally links microzooplankton biomass to their grazing pressure on phytoplankton. These patterns and relationships contribute to a stronger understanding of grazing that will inform more robust model estimates of productivity, food web dynamics, and carbon export (Rohr *et al.,* 2023).

### Seasonal variability of the microzooplankton community structure

The microzooplankton community composition and biomass on the NES exhibited consistent seasonal patterns. Dinoflagellates dominated across seasons, but winter was characterized by a majority of large dinoflagellates while small dinoflagellates were more prominent in summer. The dominance of dinoflagellates over ciliates is consistent with similar surveys of productive ecosystems in the Western Arctic Ocean (Sherr *et al.,* 2009) and the Southwest Atlantic (Sampognaro and Calliari, 2025) that also report dinoflagellates contributing a majority of the microzooplankton biomass across seasons. Strom *et al.* (2019) observed a similar shift in the size structure of the dinoflagellate community in the coastal Gulf of Alaska, with larger dinoflagellates in spring than in summer and autumn. Seasonal shifts in dinoflagellate size structure have also been observed in the Gulf of St. Lawrence, where the contribution of small dinoflagellates to the microzooplankton biomass increased in summer coinciding with a strong dominance of small phytoplankton (Tamigneaux *et al.,* 1997), as well as in the coastal Northwest Mediterranean (Calbet *et al.,* 2008). This demonstrates that our observations are generally representative across different shelf ecosystems, with a typical pattern of dominance of dinoflagellates over ciliates and a winter to summer shift from large to small dinoflagellates.

The strong seasonality on the NES provides a unique opportunity to identify the drivers of the observed patterns in community composition. Temperature has a well-known impact on body size of many organisms, including protists, and warmer temperatures are thought to favor smaller cells (Daufresne *et al.,* 2009; Liu *et al.,* 2023). Temperature impacts on protists cell size are consistent with the patterns observed here for dinoflagellates, with small dinoflagellates thriving in the warm summers on the NES, but the same is not observed for ciliates. The lack of seasonality in ciliate size structure suggests that temperature may not be playing a major role in shaping the overall microzooplankton community composition. The observation of significant grazing throughout a range of temperature conditions in the Bering Sea supports this lack of relationship between microzooplankton dynamics and temperature (Sherr *et al.,* 2013).

An alternative hypothesis is that the size structure of microzooplankton is driven by the size structure of phytoplankton. The shift in dinoflagellate size structure mirrors that of the phytoplankton community on the NES, which also undergoes a shift from large to small cells from winter to summer (Marrec *et al.,* 2021). This may suggest that microzooplankton size structure more immediately reflects the availability of prey in a suitable size class. Ciliates and dinoflagellates have distinct feeding mechanisms, which might explain their differing relationships to the size structure of the prey community. Many ciliates are suspension feeders, creating a feeding current that concentrates prey close to them before being phagocytized (Jorgensen 1983). Due to this feeding behavior, ciliates preferentially feed on prey much smaller than themselves, with a frequent predator:prey size ratio of 8:1 (Hansen *et al.,* 1994). On the other hand, dinoflagellates deploy several different feeding methods, including pallium feeding, peduncle feeding, and direct engulfment, which makes prey types available that are much larger than the dinoflagellate (Hansen and Calado, 1999). While dinoflagellates can consume a broad range of prey sizes, their median predator:prey size ratio is 1:1 (Hansen *et al.,* 1994). Ciliates’ preference for prey much smaller than themselves might allow large ciliates to persist through summer, when the prey community is dominated by small picoplankton (Fowler *et al.,* 2020) that are too small for the large dinoflagellates to feed on. These variable feeding strategies between dinoflagellates and ciliates support the differences observed in their seasonal patterns. While both ciliates and dinoflagellates both have the capability to consume a wide range of prey types and sizes, dinoflagellates wider predator:prey size spectrum makes larger phytoplankton available and results in the observed seasonal shift in dinoflagellate size structure concurrent with shifts in phytoplankton size structure.

Temperature and phytoplankton size structure were closely correlated on the NES, limiting our ability to statistically distinguish which, if either, of these parameters are driving the seasonal shift in the microzooplankton community structure. Differentiation between the effects of temperature and phytoplankton size-structure requires observations during phytoplankton size reversals, such as in summer of 2019 on the mid-shelf, where temperature and phytoplankton size structure were de-coupled. In summer of 2019 on the mid-shelf, the seasonally atypical dominance of large phytoplankton (Marrec and Menden-Deuer, 2024) occurred alongside a seasonally atypical contribution of large microzooplankton. Winter of 2020 on the outer-shelf is another example of a seasonally atypical phytoplankton size-structure, during which the phytoplankton were dominated by small cells, which is seasonally atypical. This corresponds to an atypical pattern in the microzooplankton biomass, which was comprised mostly of large ciliates. Remarkably, temperature was within the typical range for the respective season during all three outlier events, suggesting temperature did not have a causative role. These outlier events support the hypothesis that microzooplankton size structure on the NES is driven by the availability of prey in a similar size range, and not by temperature.

### Forcing factors for grazing rates

We observed that microzooplankton biomass was related to grazing pressure during winter on the NES, but not during summer. A correlation between grazer biomass and grazing rates has previously been observed in various regions including the marginal seas of China, the Western North Pacific, and the Bering Sea (Liu *et al.,* 2021, 2023). However, this relationship does not occur consistently. Previous studies have also documented a lack of relationship between microzooplankton biomass and grazing rates in other high productivity ecosystems during summertime conditions (Olson and Strom, 2002; Lawrence and Menden-Deuer, 2012; Wickham *et al.,* 2022). While it seems intuitive that grazing pressure would be closely linked to the biomass of grazers, as seen in winter, there are several possible reasons for the decoupling of microzooplankton biomass and grazing during summer. One potential explanation is a seasonal change in the dominant grazers of phytoplankton, which would dampen the link between phytoplankton and microzooplankton. Salps have been observed in high abundances during summer on the NES (Madin *et al.,* 2006), and while many mesozooplankton cannot directly consume small picoplankton, salps feed through a small mucous mesh, allowing them to consume prey that are several orders of magnitude smaller than them (Kremer and Madin, 1992; Sutherland *et al.,* 2010). It has been recently observed that salps play an important role in summer and graze a substantial amount of phytoplankton biomass on the NES (Menden-Deuer et al. in revision).

Another potential factor influencing microzooplankton biomass is the predation of microzooplankton by mesozooplankton. Crustacean zooplankton are highly abundant on the NES (Morse *et al.,* 2017) and are well known to be important grazers of microzooplankton and likely have a significant impact on their abundance and community composition, and while this trophic link is not investigated in this study, it may play a role in the inconsistent relationship between microzooplankton biomass and grazing rate (Campbell *et al.,* 2009). While in winter we observe a relationship between microzooplankton biomass and their grazing rate, a shift in the dominant type of grazer on phytoplankton from microzooplankton in the winter to gelatinous mesozooplankton in the summer may act to decouple microzooplankton biomass from their grazing impact.

During winter dinoflagellate biomass was associated with microzooplankton grazing rate. The persistent dominance of dinoflagellates throughout our samples makes it difficult to test the relationship between community composition and grazing impact. However, reduced consumption of primary production reported by Marrec and Menden-Deuer (2024) during winter of 2020 on the outer shelf coinciding with the lowest contribution of dinoflagellates to total biomass suggests that predominance of dinoflagellates results in increased grazing pressure. This occurrence of enhanced grazing when heterotrophic dinoflagellates dominate biomass has previously been observed in a nearby estuary (Lawrence and Menden-Deuer, 2012). These results suggest that dinoflagellates are the dominant microzooplankton grazer of phytoplankton during winter on the NES.

### Deviations from seasonal patterns as indicators of planktonic food web structure

Three outliers within the microzooplankton community structure were identified based on their ordination in redundancy space. Each of these outliers represent a deviation from the norm in the microzooplankton community composition, and they each correspond to concurrent deviations from baseline trends in phytoplankton dynamics, providing the opportunity to explore the link between the two. Summer of 2019 on the mid-shelf corresponds to a large bloom of *Hemiaulus*, a genus of large diatoms that were able to persist through the nutrient-depleted summertime due to their symbiosis with a nitrogen-fixing bacterium (Castillo Cieza *et al.,* 2024). This bloom led to an exceptionally high contribution of large phytoplankton to the total Chl-a concentration, which is typically dominated by small picoplankton during summer. It was also accompanied by elevated primary production (Castillo Cieza *et al.,* 2024), microzooplankton grazing, and trophic transfer efficiency (Marrec and Menden-Deuer, 2024). During this bloom, the microzooplankton community was dominated by large dinoflagellates, a population likely supported by the high abundances of *Hemiaulus*, since large dinoflagellates are well known to feed on large diatoms (Sherr and Sherr, 2007).

Summer of 2018 on the outer shelf was another high microzooplankton biomass event that was also accompanied by elevated net community production and gross oxygen production, similar to that observed during the 2019 *Hemiaulus* bloom (Castillo Cieza *et al.,* 2024). However, the phytoplankton community structure did not show dominance of large phytoplankton cells in summer 2018 on the outer-shelf, suggesting that an alternative mechanism than the dominance of large phytoplankton cells was driving the high production and the elevated microzooplankton biomass observed. Although the microzooplankton community deviated from its typical seasonal pattern at this time of high productivity, grazing pressure remained typically low.

Winter of 2020 on the outer shelf was characterized by a near-average total microzooplankton biomass, but the highest proportion of ciliates was observed on the shelf. During this period, the phytoplankton size structure was seasonally reversed, and while winter is usually dominated by large phytoplankton, a higher contribution of small phytoplankton was observed, coinciding with a low trophic transfer efficiency from phytoplankton to microzooplankton. (Marrec and Menden-Deuer, 2024). The much larger predator:prey size ratio for ciliates than dinoflagellates in the presence of high concentrations of small phytoplankton may have allowed the ciliates to out-compete large dinoflagellates that usually make up the majority of the microzooplankton community during winter, supporting the hypothesis that the microzooplankton size structure is driven by the phytoplankton size structure.

Two of these events support the hypothesis that microzooplankton community structure is driven by phytoplankton size structure, with clear impacts in terms of microzooplankton grazing and trophic transfer efficiency within the plankton food web. Summer of 2018 on the mid-shelf represents an outlier with no change in phytoplankton size structure, but with elevated productivity. Outliers in the microzooplankton community occurred alongside atypical phytoplankton conditions, either in size structure or productivity, emphasizing the important link between the two. This further suggests that they are genuine outliers rather than methodological artifacts, and that the present dataset establishes a baseline against which deviations can be detected, and from which hypotheses about the drivers of grazing can be developed.

## CONCLUSIONS

Microzooplankton biomass and community composition are seasonal on the NES, with higher total biomass in winter than summer. Dinoflagellates have a distinct seasonal size structure with a majority of large dinoflagellates in winter and small dinoflagellates in summer, while ciliate size structure and biomass do not exhibit the same seasonality. Grazing rates were significantly correlated with total microzooplankton biomass in winter only, which may suggest an alternative loss process for phytoplankton is acting to decouple microzooplankton biomass from their grazing rates, or an alternative control on microzooplankton biomass and grazing pressure due to predation by mesozooplankton. Outliers of microzooplankton community composition correspond to outliers in both phytoplankton community structure and trophic transfer efficiency (Marrec and Menden-Deuer, 2024), suggesting that microzooplankton biomass and grazing impact reflect available prey size structure.

Microzooplankton are critical components of many biogeochemical cycles in the ocean, and these observations allow us to better understand what mediates their impact across variable environmental and biological conditions. Understanding these large-scale patterns of the microzooplankton community, how they are linked to phytoplankton dynamics, and how they impact grazing rates shed light on the role that microzooplankton play in marine food webs. Grazing by microzooplankton is an essential pathway for carbon and energy transfer at the base of marine food webs, with large potential for implications at higher trophic levels. Our results provide a baseline for the structure of microzooplankton communities on the NES and document the tight linkage between the structure (e.g. community compositions) and function (e.g. trophic transfer) of planktonic food webs. Sustained observations within the NES-LTER will allow for a better understanding of how long-term environmental changes associated with climate and anthropogenic perturbations will impact the structure and function of planktonic food webs in the future.

## Supplementary Material

MZBiomass_Supplemental_FINAL_fbag018

## Data Availability

The data that support the findings of this study are openly available in Environmental Data Initiative at: https://doi.org/10.6073/pasta/2d1bf323b99bc35e5dbedfd37eed939a (microzooplankton biomass); https://doi.org/10.6073/pasta/a8170b4f30fec183592ea7868d7bc1d4 (size-fractionated Chl a); https://doi.org/10.6073/pasta/b3366e0d4206a401b415953ed3e60389 (microzooplankton grazing rates); https://doi.org/10.6073/pasta/abc6c4301cc3e0317e68ca118711f682 (nutrient data).
