# Supplementary material for: Seasonal variability of the microzooplankton biomass and community composition on the Northeast US Shelf
Source: J Plankton Res. 2026 Mar 28;48(2):fbag018. doi: 10.1093/plankt/fbag018 (PMC13031981; doi:10.1093/plankt/fbag018)
Supplement: MZBiomass_Supplemental_FINAL_fbag018 [file mzbiomass_supplemental_final_fbag018.docx]

Supplemental Information for:

**Seasonal Variability of the Microzooplankton Biomass and Community Composition on the Northeast US Shelf**

Frankie Lopez, Pierre Marrec, Susanne Menden-Deuer

Graduate School of Oceanography, University of Rhode Island, Narragansett, RI, 02882, USA

**List of Supplemental Figures and Tables**

**Figure S1.** Correlation heatmap for all environmental and microzooplankton variables. Microzooplankton biomass variables (small dinos, large dinos, small ciliates, and large ciliates) are biomass of each group relative to the total biomass of all four groups. Environmental variables are normalized by z-score. Positive correlations are red and negative correlations are blue. The Pearson’s correlation coefficient (r) for each relationship is stated within each cell, and the values are bold if the correlation is significant with a p-value*<*0.05.

**Figure S2.** Correlation heatmap for all measures of microzooplankton biomass with rates of phytoplankton growth and mortality due to microzooplankton grazing for both seasons together (a), winter only (b), and summer only (c). Positive correlations are red and negative correlations are blue. The Pearson’s correlation coefficient (r) for each relationship is stated within each cell, and the values are bold if the correlation is significant with a p-value*<*0.05.

**Table S1.** Biomass of dinoflagellates in the genus *Tripos* (μg C L^−1^).

**Table S2.** Mean and standard deviation for all environmental parameters for each season and region with outlier events excluded from calculation of averages.

**Table S3.** Environmental conditions for the three outlier events identified in Figure 2.


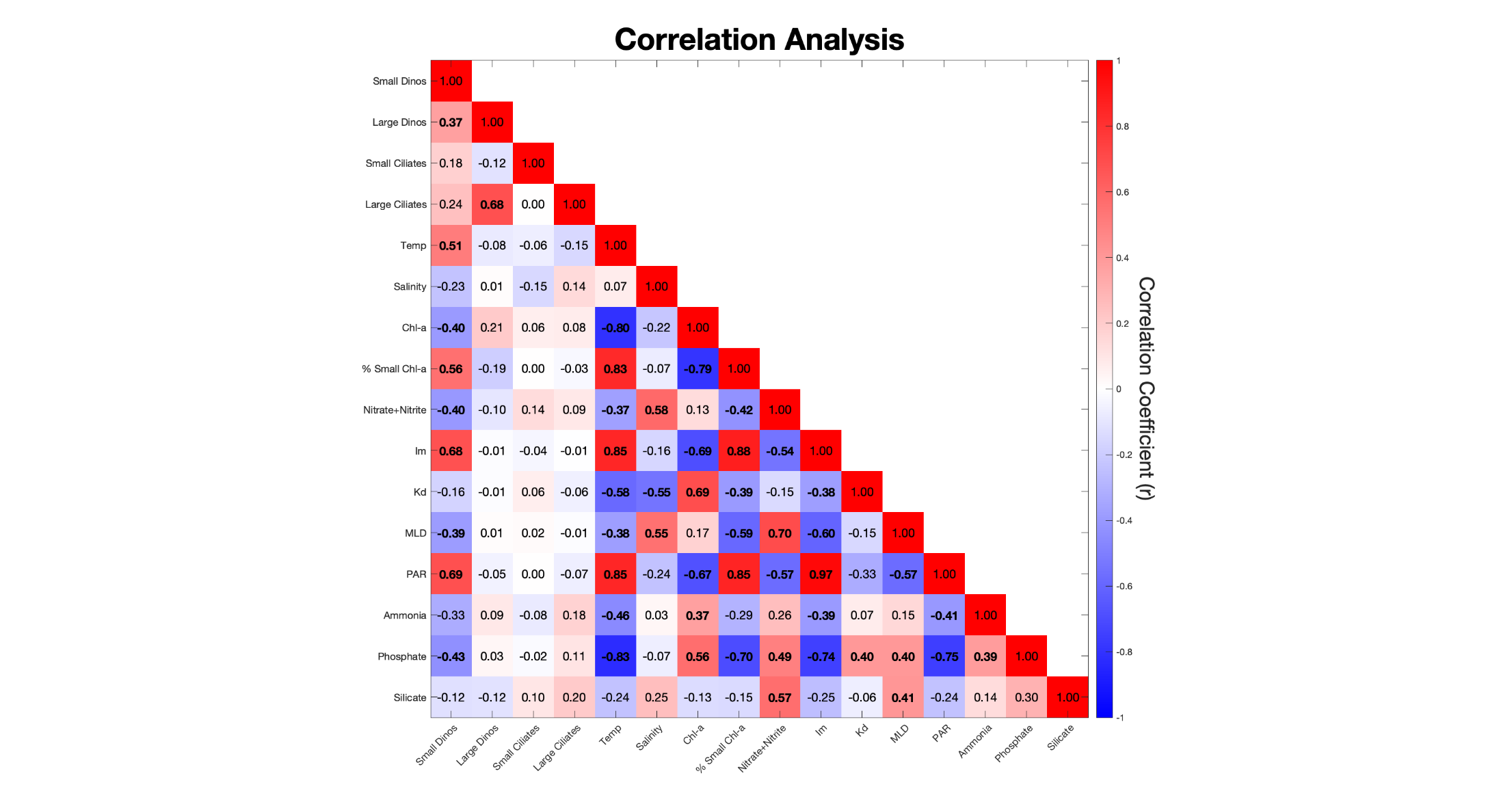


**Figure S1.** Correlation heatmap for all environmental and microzooplankton variables. Microzooplankton biomass variables (small dinos, large dinos, small ciliates, and large ciliates) are biomass of each group relative to the total biomass of all four groups. Environmental variables are normalized by z-score. Positive correlations are red and negative correlations are blue. The Pearson’s correlation coefficient (r) for each relationship is stated within each cell, and the values are bold if the correlation is significant with a p-value<0.05.


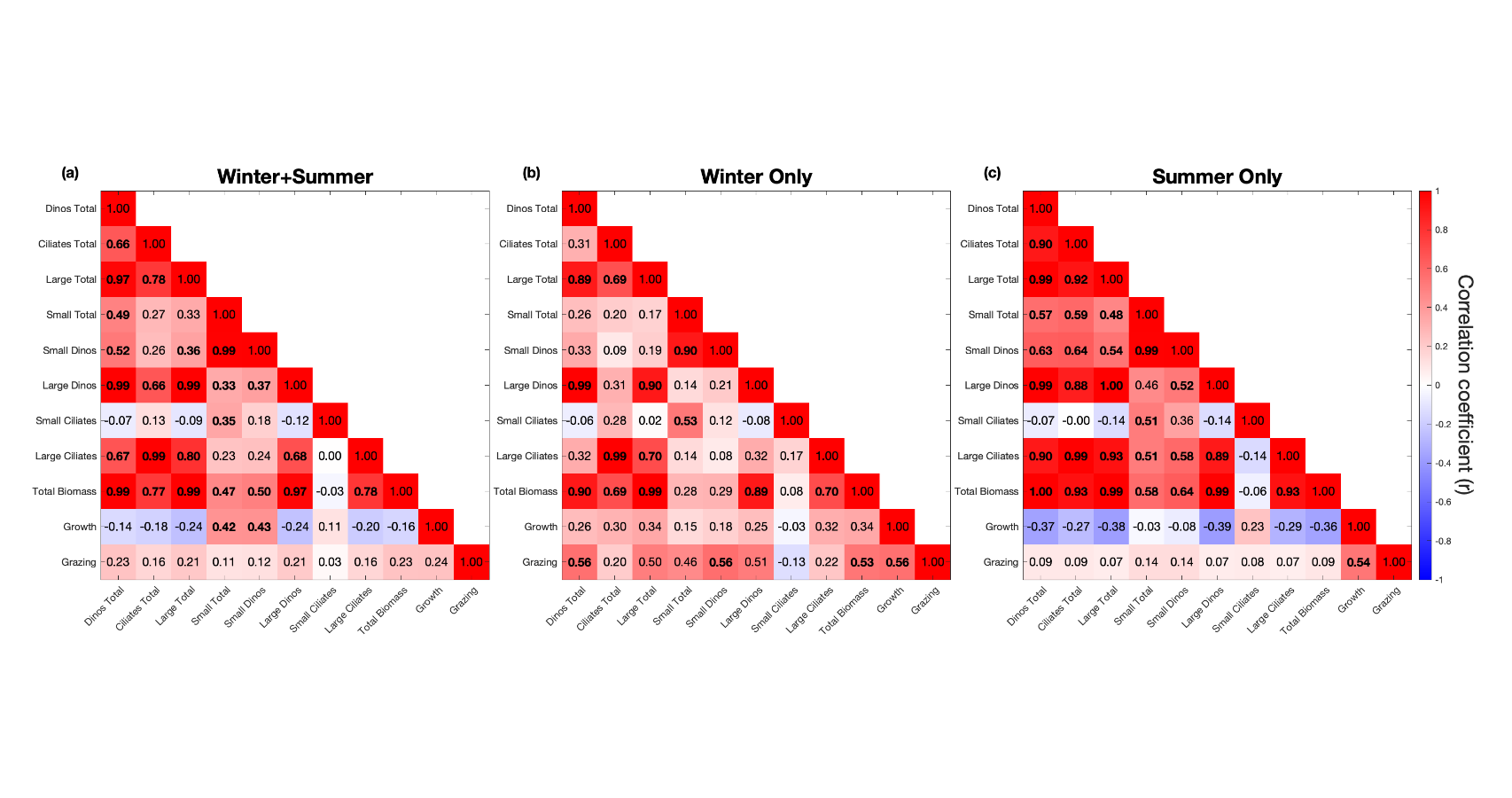


**Figure S2.** Correlation heatmap for all measures of microzooplankton biomass with rates of phytoplankton growth and mortality due to microzooplankton grazing for both seasons together (a), winter only (b), and summer only (c). Positive correlations are red and negative correlations are blue. The Pearson’s correlation coefficient (r) for each relationship is stated within each cell, and the values are bold if the correlation is significant with a p-value<0.05.

**Table S1*.*** Biomass of dinoflagellates in the genus *Tripos* (μg C L^−1^).

|  | Winter | | | Summer | | |
| --- | --- | --- | --- | --- | --- | --- |
|  | Inner-Shelf | Mid-Shelf | Outer-Shelf | Inner-Shelf | Mid-Shelf | Outer-Shelf |
| 2018 | 1 | 5 | 5 | 0 | 0 | 0 |
| 2019 | 0 | 1 | 6 | 0 | 5 | 0 |
| 2020 | 0 | 0 | 0 | 0 | 0 | 0 |
| 2021 | 2 | 3 | 0 | 1 | 1 | 0 |
| 2022 | 0 | 2 | 0 | 2 | 10 | 0 |
| 2023 | 0 | 0 | 0 | 21 | 0 | 0 |

**Table S2.** Mean and standard deviation for all environmental parameters for each season and region with outlier events excluded from calculation of averages.

|  | Winter | | | | | | | | | Summer | | | | | | | | |
| --- | --- | --- | --- | --- | --- | --- | --- | --- | --- | --- | --- | --- | --- | --- | --- | --- | --- | --- |
|  | Inner-Shelf | | | Mid-Shelf | | | Outer-Shelf | | | Inner-Shelf | | | Mid-Shelf | | | Outer-Shelf | | |
| Temperature  (°C) | 4.32 | ± | 1.46 | 6.19 | ± | 1.91 | 11.89 | ± | 3.08 | 20.78 | ± | 1.25 | 22.50 | ± | 1.28 | 25.50 | ± | 1.84 |
| Salinity | 32.40 | ± | 0.28 | 32.72 | ± | 0.31 | 34.67 | ± | 0.90 | 31.85 | ± | 0.34 | 32.18 | ± | 0.68 | 34.09 | ± | 0.47 |
| Chl-a  (µg L^-1^) | 3.61 | ± | 1.14 | 2.54 | ± | 1.24 | 1.87 | ± | 1.04 | 0.92 | ± | 0.29 | 0.40 | ± | 0.09 | 0.11 | ± | 0.02 |
| Small Chl-a (%) | 22 | ± | 10 | 26 | ± | 16 | 42 | ± | 16 | 78 | ± | 16 | 78 | ± | 9 | 88 | ± | 15 |
| Nitrate+Nitrite (µmol L^-1^) | 0.40 | ± | 0.88 | 1.65 | ± | 0.73 | 4.14 | ± | 1.72 | 0.00 | ± | 0.00 | 0.00 | ± | 0.00 | 0.06 | ± | 0.13 |
| Ammonium (µmol L^-1^) | 0.32 | ± | 0.38 | 0.45 | ± | 0.52 | 0.27 | ± | 0.52 | 0.05 | ± | 0.06 | 0.05 | ± | 0.05 | 0.11 | ± | 0.09 |
| Phosphate (µmol L^-1^) | 0.36 | ± | 0.13 | 0.39 | ± | 0.12 | 0.30 | ± | 0.14 | 0.16 | ± | 0.07 | 0.06 | ± | 0.07 | 0.00 | ± | 0.01 |
| Silicate (µmol L^-1^) | 0.57 | ± | 0.78 | 1.87 | ± | 1.12 | 2.16 | ± | 0.75 | 1.31 | ± | 1.20 | 1.07 | ± | 0.63 | 0.48 | ± | 0.22 |
| MLD  (m) | 15.9 | ± | 8.0 | 48.0 | ± | 24.1 | 100.1 | ± | 45.5 | 5.7 | ± | 1.6 | 7.9 | ± | 3.1 | 12.9 | ± | 7.1 |
| K_d_  (m^-1^) | 0.32 | ± | 0.12 | 0.18 | ± | 0.03 | 0.12 | ± | 0.03 | 0.21 | ± | 0.04 | 0.14 | ± | 0.01 | 0.07 | ± | 0.01 |
| PAR  (mol photons m^-2^ d^-1^) | 17.5 | ± | 3.4 | 17.1 | ± | 3.8 | 16.3 | ± | 4.9 | 47.7 | ± | 9.8 | 50.0 | ± | 8.3 | 49.7 | ± | 6.8 |
| I_m_  (mol photons m^-2^ d^-1^) | 3.2 | ± | 1.4 | 1.7 | ± | 0.7 | 2.2 | ± | 1.3 | 28.1 | ± | 7.3 | 29.5 | ± | 7.8 | 30.6 | ± | 8.4 |

**Table S3.** Environmental conditions for the three outlier events identified in Figure 2.

|  | Summer 2018  Outer-Shelf | Summer 2019 Mid-Shelf | Winter 2020  Outer-Shelf |
| --- | --- | --- | --- |
| Temperature  (°C) | 23.06 | 24.49 | 12.50 |
| Salinity | 34.62 | 31.90 | 35.00 |
| Chl-a  (µg L^-1^) | 0.27 | 3.07 | 1.06 |
| Small Chl-a (%) | 59 | 6 | 73 |
| Nitrate+Nitrite (µmol L^-1^) | 0.00 | 0.00 | 5.79 |
| Ammonium (µmol L^-1^) | 0.00 | 0.23 | 0.70 |
| Phosphate (µmol L^-1^) | 0.09 | 0.00 | 0.38 |
| Silicate (µmol L^-1^) | 0.78 | 0.00 | 3.20 |
| MLD  (m) | 24.2 | 11.1 | 12.1 |
| K_d_  (m^-1^) | 0.02 | 0.17 | 0.10 |
| PAR  (mol photons m^-2^ d^-1^) | 50.3 | 40.7 | 16.0 |
| I_m_  (mol photons m^-2^ d^-1^) | 39.0 | 18.0 | 9.2 |
